# Haptoglobin-α1, -α2, vitamin D-binding protein and apolipoprotein C-III as predictors of etanercept drug response in rheumatoid arthritis

**DOI:** 10.1186/s13075-015-0553-1

**Published:** 2015-03-06

**Authors:** Sabine Blaschke, Kathinka Rinke, Michael Maring, Thomas Flad, Susann Patschan, Olaf Jahn, Claudia A Mueller, Gerhard A Mueller, Hassan Dihazi

**Affiliations:** Department of Nephrology and Rheumatology, University Medical Center of Göttingen, Robert-Koch Straße 40, 37075 Göttingen, Germany; PANATecs GmbH, Inselwiesenstr. 10, 74076 Heilbronn, Germany; Proteomics Group, Max-Planck-Institute of Experimental Medicine, Hermann-Rein Str. 3, 37075 Göttingen, Germany; Section for Transplantation Immunology and Immunohematology, Waldhörnlestr. 22, 72072 Tübingen, Germany

## Abstract

**Introduction:**

The introduction of tumor necrosis factor-alpha (TNF-α) antagonists has substantially improved patient’s clinical outcome in rheumatoid arthritis (RA). However, nearly 20% to 40% of RA patients do not respond to anti-TNF-α treatment strategies. To identify valid predictors of TNF-α antagonist response in RA, serum proteome profiles from responders (R) and non-responders (NR) to etanercept, a soluble recombinant TNF-α receptor/IgG Fc fusion protein receptor, were compared in a prospective cohort study.

**Methods:**

In this clinical study 50 RA patients with inadequate response to conventional DMARDs were included and treated with etanercept. The primary efficacy endpoint was response according to the European League against Rheumatism (EULAR) improvement criteria. Serum samples collected prior to initiation and after six months of etanercept therapy were cleared of the most abundant major proteins by immunoaffinity chromatography. After separation by two-dimensional differential gel electrophoresis (2D-DIGE) and identification by mass spectrometry (MS) data were validated by Western blot analysis.

**Results:**

After six months of etanercept treatment 62% (n = 31) of RA patients achieved response. Haptoglobin-α1 (Hp-α1) and -α2 (Hp-α2) and vitamin D-binding protein (VDBP) were found to be significantly upregulated in responder sera (*P* ≤0.02) at study entry. In contrast, apolipoprotein C-III (ApoC-III) showed significantly higher levels in non-responders (*P* = 0.0162). At study end ApoA-II, Hp-α1, Hp-α2 and VDBP were identified to be expressed at significantly higher levels (*P* <0.05) in responder sera.

**Conclusions:**

By application of clinical proteomics in immunodepleted sera we could identify and validate for the first time Hp-α1, -α2, VDBP and ApoC-III as potential biomarkers for prediction of etanercept drug response in RA.

**Electronic supplementary material:**

The online version of this article (doi:10.1186/s13075-015-0553-1) contains supplementary material, which is available to authorized users.

## Introduction

Rheumatoid arthritis (RA) is a chronic inflammatory disease of still unknown etiology with a prevalence of about 1% in the western population leading to progressive joint deformities by cartilage destruction and bone erosion [1]. For the pathogenesis of RA, proinflammatory cytokines – especially tumor necrosis factor alpha (TNFα) – were shown to play one of the most important roles in RA pathogenesis by stimulation of matrix metalloproteinase and proteolytic enzyme release from synoviocytes leading to local cartilage degradation and demineralization of bone in affected joints [2]. The course of the disease is quite variable; however, about 10% of patients experience progressive disease leading to marked joint deformities and disability.

Conventional therapeutic approaches in RA are directed at a nonspecific suppression of the inflammatory process by so-called disease-modifying antirheumatic drugs (DMARDs), such as the gold standard methotrexate and leflunomide [3].

The introduction of biologic DMARD therapy targeting proinflammatory cytokines, especially TNFα antagonists, has substantially improved patient’s clinical outcome in RA. Different TNFα inhibitors have so far been approved for the treatment of RA. Efficacy and safety of the TNFα antagonist etanercept [4], a soluble recombinant TNFα receptor/IgG Fc fusion protein receptor, have been demonstrated in randomized and placebo-controlled studies. Results of subsequent clinical trials suggest that concomitant methotrexate therapy may enhance its efficacy [5]. However, despite these beneficial effects in RA patients’ treatment outcome, high therapeutic costs and adverse drug reactions – such as severe infections, congestive heart failure, and so forth [6] – limit a broad application of TNFα antagonists in RA.

In addition, 20 to 40% of RA patients were previously shown not to respond to anti-TNFα treatment strategies [5,7]. Treatment failure may manifest as primary nonresponse or secondary loss of response. Several factors may account for the lack of efficacy, including different pharmacokinetics of TNFα inhibitors, development of anti-TNFα inhibitor antibodies, pharmacogenetics and inadequate concomitant DMARD therapy. However, previous studies analyzing demographic factors [8], baseline disease characteristics [9] or pharmacogenetic factors [10] could not so far identify valid predictors of response to anti-TNFα therapy in RA.

Recently, proteomic technologies – in particular, capillary electrophoresis or liquid chromatography coupled to electrospray ionization mass spectrometry (MS) as well as surface enhanced laser desorption/ionization time-of-flight MS together with bioinformatics tools – have successfully been employed for identification of diagnostic markers and therapeutic targets even in highly complex body fluids [11,12] as well as for identification of drug-modulated targets and response predictors [13]. It is thus hypothesized that analysis of TNFα antagonist-modulated alterations of the molecular circuitry by serum proteomic profiling may help to identify response predictors for TNFα antagonist therapy in RA.

In this prospective clinical cohort study, proteome profiles of serum samples were compared between responders and nonresponders to etanercept treatment in RA patients to identify potential predictors of drug response.

## Methods

### Patients and samples

A prospective clinical study was performed at the University Medical Center Goettingen, Germany. The study was conducted in compliance with the principles laid down in the latest version of the Declaration of Helsinki and was finally approved by the local Institutional Review Board at the University Medical Center Goettingen (No. 14/12/2007).

All included patients (*n* = 50) met the criteria for RA established by the American College of Rheumatology [14] of more than 6 months’ disease duration. All RA patients had active disease defined by three or more tender joints, three or more swollen joints, physician and patient assessment of pain of at least 40 mm or more on a 10 cm visual analogue scale and morning stiffness for >1 hour, with an elevated erythrocyte sedimentation rate (>28 mm/hour) or C-reactive protein (CRP >8 mg/l). Furthermore, all RA patients had inadequate response to standard immunosuppressive therapy with methotrexate or leflunomide. Exclusion criteria comprised all contraindications against etanercept. After evaluation of all inclusion and exclusion criteria at the screening visit (V1) and written informed consent, RA patients were enrolled in the study and assigned to receive etanercept treatment (50 mg subcutaneously once per week) as TNFα antagonist therapy at the baseline visit (V2). At each visit and at trial week 4 (V3), week 8 (V4), week 12 (V5) and week 24 (V6), clinical disease activity (Disease Activity Score in 28 joints (DAS28)) [15], physical function (Health Assessment Questionnaire score) [16], vital signs and laboratory parameters including hematology, clinical chemistry and immunologic parameters as well as CRP, erythrocyte sedimentation rate, rheumatoid factor and anti-cyclic citrullinated peptide antibodies were assessed in the local laboratory. After 6 months of etanercept treatment, response was assessed according to the European League against Rheumatism improvement criteria [17]. A major (good or moderate) response was defined as a decrease of DAS28 ≥ 1.2. Baseline characteristics of all RA patients also including cardiovascular risk factors (arterial hypertension, diabetes, tobacco use, hyperlipoproteinemia, obesity, family history) are summarized in Table 1.

**Table 1:** **Baseline characteristics of rheumatoid arthritis patients at study entry**

**Patients**	**Responder**	**Nonresponder**	***P*** **value**
Number of patients	31	19	
Sex (male:female)	7:24	4:15	0.61
Mean (range) age (years)	55.8 (41 to 75)	55.9 (40 to 71)	0.97
Cardiovascular risk factors (≥1, % positive)	51.1	65.4	0.12
IgM-RF (% positive)	54.8	31.6	0.17
Anti-CCP (% positive)	64.5	31.6	0.15
Mean (standard deviation) DAS28	4.82 (±1.19)	5.30 (±1.55)	0.22
Mean (range) CRP (mg/l)	10.9 (2.0 to 114.5)	11.4 (2.0 to 80.7)	0.93
Mean (range) disease duration (years)	6.92 (2 to 29)	5.4 (1 to 19)	0.84
Therapy			
Corticosteroids	23	11	
DMARDs			
Methotrexate	24	15	
Leflunomide	7	4	

Anti-CCP, anti-cyclic citrullinated peptide antibodies; CRP, C-reactive protein; DAS28, Disease activity score in 28 joints at study visit V1; DMARD, disease-modifying antirheumatic drug; RF, rheumatoid factor.

For clinical proteomics, sera (10 ml) were collected from each RA patient prior to the initiation of TNFα antagonist therapy (V1 and V2, pre-treatment samples) and during the follow-up period at trial week 12 (V5) and trial week 24 (V6) (post-treatment samples). Samples were stored at –80°C until analysis.

### Serum depletion and protein precipitation

For clinical proteomics, 10 ml serum samples were taken from RA patients at visits V1, V5 and V6. Sera were then subjected to high-performance liquid chromatography-based immunodepletion for high abundant proteins using Human-14® immunoaffinity columns (Agilent, Boeblingen, Germany). The depletion of high abundant proteins from sera was carried out according to the manufacturer’s protocol. Subsequently, 6 M urea was added and the samples containing the low abundant protein fractions were concentrated using Vivaspin 4® (Sartorius Stedim, Goettingen, Germany) ultrafiltration spin columns. Samples were mixed with ice-cold precipitation solution (20% trichlororacetic acid in acetone) at a ratio of 1:3. After centrifugation the protein pellets were washed with ice-cold acetone and dried for 5 minutes. The total protein concentration was estimated using the Bradford method with bovine serum albumin (1 mg/ml) as the standard [18] using the LAMBDA™ 25 spectrophotometer (PerkinElmer, Waltham, Massachusetts, USA).

### Two-dimensional gel electrophoresis

For large-scale two-dimensional gel electrophoresis (2-DE), immobilized pH gradient strips (11 cm, pI 3 to 10, pl 5 to 8) were passively rehydrated in 185 μl solution containing 150 μg protein in a rehydration buffer (8 M urea, 1% (w/v) 3-((3-cholamidopropyl)-dimethylammonio)-1-propane sulfonate (CHAPS), 1% dithiothreitol (DTT), 0.2% ampholytes, and a trace of bromophenol blue) for 12 hours. The isoelectric focusing step was performed on the PROTEAN® IEF Cell (Bio-Rad, Hercules, California, USA). Temperature controlled at 20°C, the voltage was set to 500 V for 1 hour, increased to 1,000 V for 1 hour and 2,000 V for 1 hour, and left at 8,000 V until a total of 50,000 V hours was reached. Prior to SDS-PAGE, the immobilized pH gradient strips were each reduced for 20 minutes at room temperature in SDS equilibration buffer containing 6 M urea, 30% glycerol, 2% SDS 0.05 M Tris–HCl, and 2% DTT on a rocking table. The strips were subsequently alkylated in the same solution with 2.5% iodacetamide substituted for DTT, and a trace of bromophenol blue. For the SDS-PAGE, 12% BisTris Criterion® precast gels (Bio-Rad) were used according to the manufacturer’s instructions. The gels were run at 150 V for 10 minutes following by 200 V until the bromophenol blue dye front had reached the bottom of the gel.

### Gel staining

For image analysis, 2-DE gels were fixed in a solution containing 50% methanol and 12% acetic acid overnight and stained with Flamingo® fluorescent gel stain (Bio-Rad) for 5 hours. Gels were then scanned at 50 μm resolution on a Fuji FLA-5100 scanner (Fuji Photo, Kanagawa, Japan). Digitalized images were analyzed using Delta 2D 3.4 (Decodon, Greifswald, Germany). 2-DE gels were post-stained with colloidal Coomassie® (Roti-Blue; Roth, Karlsruhe, Germany) overnight to enable manual spot picking for protein identification.

### Protein CyDyes minimal labeling and two-dimensional differential gel electrophoresis

To assure high data quality for two-dimensional differential gel electrophoresis (2D-DIGE), four biological replicates consisting of sera from four patients per group were collected. Serum depletion and protein precipitation were performed as described above. The resulting pellet was solubilized in labeling buffer (30 mM Tris–HCl pH 8.5, 9.5 M urea, 2% CHAPS, 10 mM phenylmethylsulfonylfluoride) and centrifuged (5 minutes, 13,000 × *g*), and the protein concentration of the supernatant was determined. Protein samples were labeled with CyDyes as described previously [19]. For minimal labeling, 400 pmol amine-reactive cyanine dyes Cy3 and Cy5 were added to 50 μg proteins from responders and nonresponders, respectively. The labeling reaction was carried out at 4°C in the dark for 30 minutes and the reaction was terminated by addition of 10 nmol lysine at 4°C in the dark for 10 minutes. Equal volumes of 2× sample buffer (30 mM Tris–HCl pH 8.5, 9.5 M urea, 2% CHAPS, 10 mM phenylmethylsulfonylfluoride, 130 mM DTT and 2% ampholytes 3-10) were added. To avoid dye-specific protein labeling, every pair of protein samples from two independent depleted serum samples from the same patient were processed in duplicate while swapping the dyes. Thereby four replicate gels per patient serum were obtained. Then 50 μg internal standard consisting of a mixture of the two samples (responder and nonresponder) under investigation were labeled with 400 pmol Cy2 and included on all gels to facilitate gel matching. The three differentially labeled fractions were pooled. Rehydration buffer (8 M urea, 1% CHAPS, 13 mM DTT and 1% ampholytes 3-10) was added to make up the volume to 185 μl prior to isoelectric focusing. The 2-DE was performed as described previously [19]. The CyDye-labeled protein gels were scanned at 50 μm resolution on a Fuji FLA-5100 scanner with excitation light at long pass 473 nm (Cy2), 575 nm (Cy3) and 635 nm (Cy5). Fluorescent images were acquired in 16-bit TIFF file format. Spot matching across gels and normalization based on the internal standard was performed with Delta2D 3.4 software. Statistical significance of protein regulation as assessed by Student’s *t* test was assumed for *P* <0.01. After post-staining with Coomassie®, differentially regulated proteins were excised and processed for identification by MS.

### In-gel digestion and mass spectrometry analysis

Manually excised gel plugs were subjected to an automated platform for the identification of gel-separated proteins as described recently [20]. An Ultraflex MALDI-TOF-TOF mass spectrometer (Bruker Daltonik, Bremen, Germany) was used to acquire both peptide mass fingerprinting and fragment ion spectra, resulting in confident protein identifications based on peptide mass and sequence information. Database searches in the Swiss-Prot primary sequence database restricted to the taxonomy *Homo sapiens* were performed using the MASCOT Software 2.2 (Matrix Science, London, UK). Carboxamidomethylation of Cys residues was specified as fixed modification and oxidation of Met residues as variable modifications. One trypsin-missed cleavage was allowed. Mass tolerances were set to 100 ppm for peptide mass fingerprinting searches and to 100 ppm (precursor ions) and 0.7 Da (fragment ions) for MS/MS ion searches. The minimal requirement for accepting a protein as identified was at least one peptide sequence match above identity threshold in addition to at least 20% sequence coverage in the peptide mass fingerprinting.

### Western blot analysis

Validation of the 2-DE data was carried out using western blot (WB) analysis. To assure the reproducibility of the WB analysis, at least three experimental replicates from each patient’s serum were performed. Proteins (40 μg) were separated by SDS-PAGE and transferred to Hybond® ECL nitrocellulose membrane (GE Healthcare, Freiburg, Germany). Immunodetection was performed as described previously [21]. Briefly, membranes were blocked in 5% milk for 2 hours at room temperature, followed by overnight incubation at 4°C with diluted specific primary antibody including polyclonal rabbit anti-human haptoglobin (Hp; 1:1,000), rabbit anti-human apolipoprotein (Apo) C-III antibody (Genway, San Diego, CA, USA), rabbit anti-human ApoA-II antibody (Genway) or a rabbit anti-human vitamin D-binding protein antibody (Abcam, Cambridge, UK).

After washing three times in Tris-buffered saline–Tween buffer, nitrocellulose membranes were incubated with the corresponding secondary antibody (Alexa Fluor 647 goat anti-mouse IgG antibody or Alexa Fluor 647 goat anti-rabbit IgG, 1:2,000; Molecular Probes, Darmstadt, Germany). Air-dried blot membranes were then scanned at 50 μm resolution on a Fuji FLA-5100 scanner with single laser-emitting excitation light at 635 nm.

### Two-dimensional western blot

Proteins (150 μg) were separated by isoelectric focusing and SDS-PAGE as described above and transferred to Hybond® ECL nitrocellulose membrane. Immunodetection was performed as described above. Mouse monoclonal anti-vitamin D-binding protein (1:1,000; Abcam) was used as primary antibody. Molecular Probes Alexa Fluor 647 goat anti-mouse IgG antibody (1:2,000) was used as secondary antibody.

### Statistical analysis

All blots were quantified using ImageJ software (Open Access Bioimaging Software, NIH, Bethesda, Maryland, USA). The data were compiled with the software package GraphPad Prism (version 4; La Jolla, California, USA). Statistical differences between patient groups (responders and nonresponders) were calculated by Student’s t test for paired and unpaired samples. Results are presented as the mean ± standard deviation of at least three independent experiments. Differences were considered statistically significant when *P* <0.05.

## Results

### Clinical outcome of etanercept treatment in RA patients

**Figure 1:**
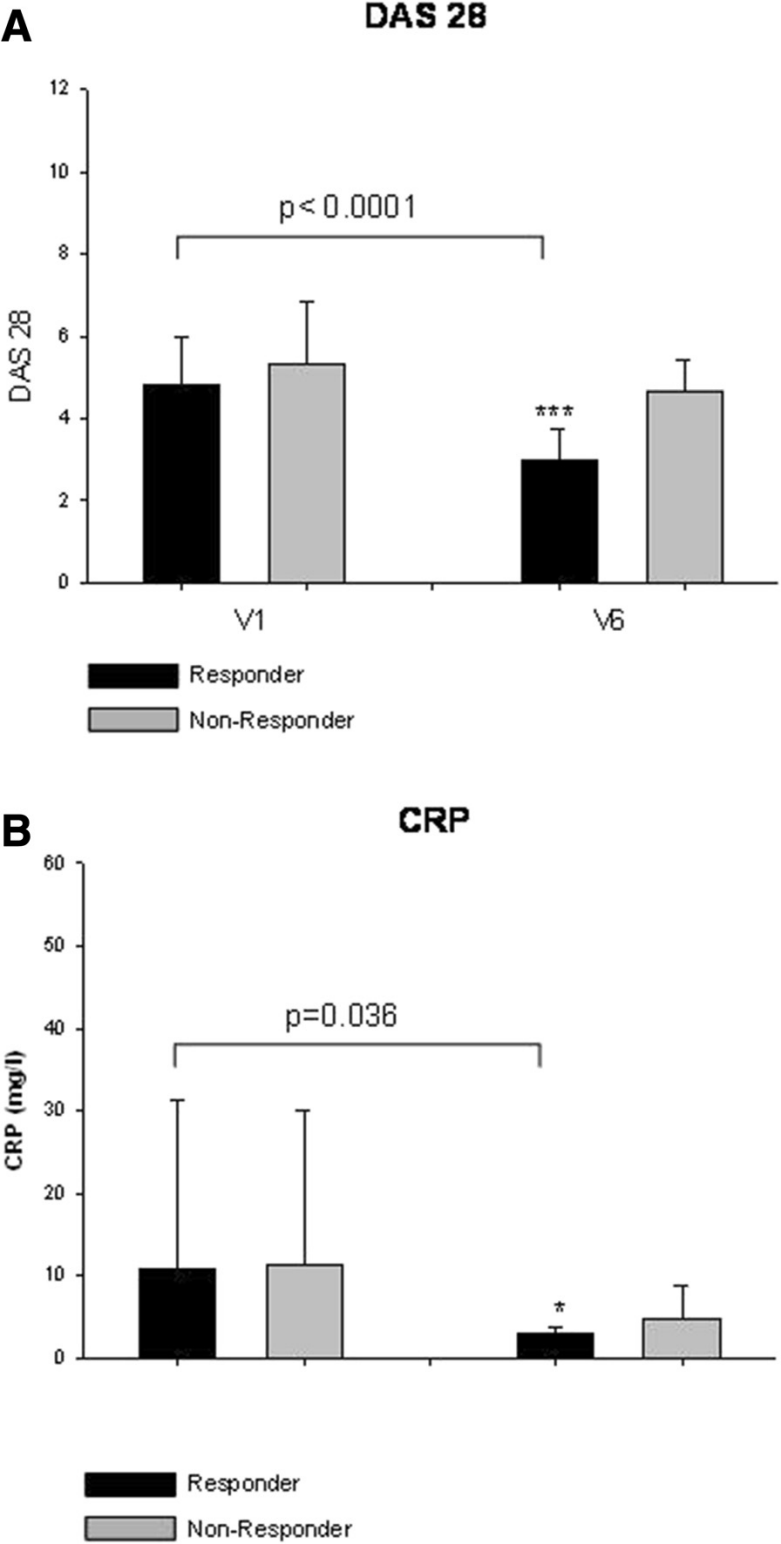
**Clinical outcome of etanercept treatment in rheumatoid arthritis patients.** In this cohort study, patients with rheumatoid arthritis (*n* = 50) and inadequate response to conventional disease-modifying antirheumatic drugs were included. All patients were subjected to etanercept (50 mg once per week) therapy and evaluated according to European League against Rheumatism improvement criteria after 6 months of therapy. **(A)** Disease Activity Score in 28 joints (DAS28) scores compared between responders (R) and nonresponders (NR) at baseline visit (V1) and at the end of study (V6). **(B)** Furthermore, C-reactive protein (CRP) levels were measured and compared at V1 and V6.

In this prospective cohort study, 50 RA patients with high disease activity and inadequate response to standard DMARD therapy were included. After 6 months of etanercept treatment (50 mg per week), 62% (*n* = 31) of RA patients were judged as responders and 38% (*n* = 19) as nonresponders to etanercept treatment. By comparison of DAS28 scores between the baseline visit (V1) and end of study (V6), these results were also reflected by a significant reduction of DAS28 scores within the responder group (*P* <0.0001) (Figure 1A). Furthermore, CRP and interleukin (IL)-6 (data not shown) serum levels were significantly reduced after 6 months of etanercept therapy in responders (*P* = 0.036) (Figure 1B). No significant changes were observed in the nonresponder group for DAS28, CRP and IL-6 (data not shown) serum levels.

### Comparative analysis of differentially expressed proteins in sera of responders and nonresponders to etanercept treatment in RA patients

**Figure 2:**
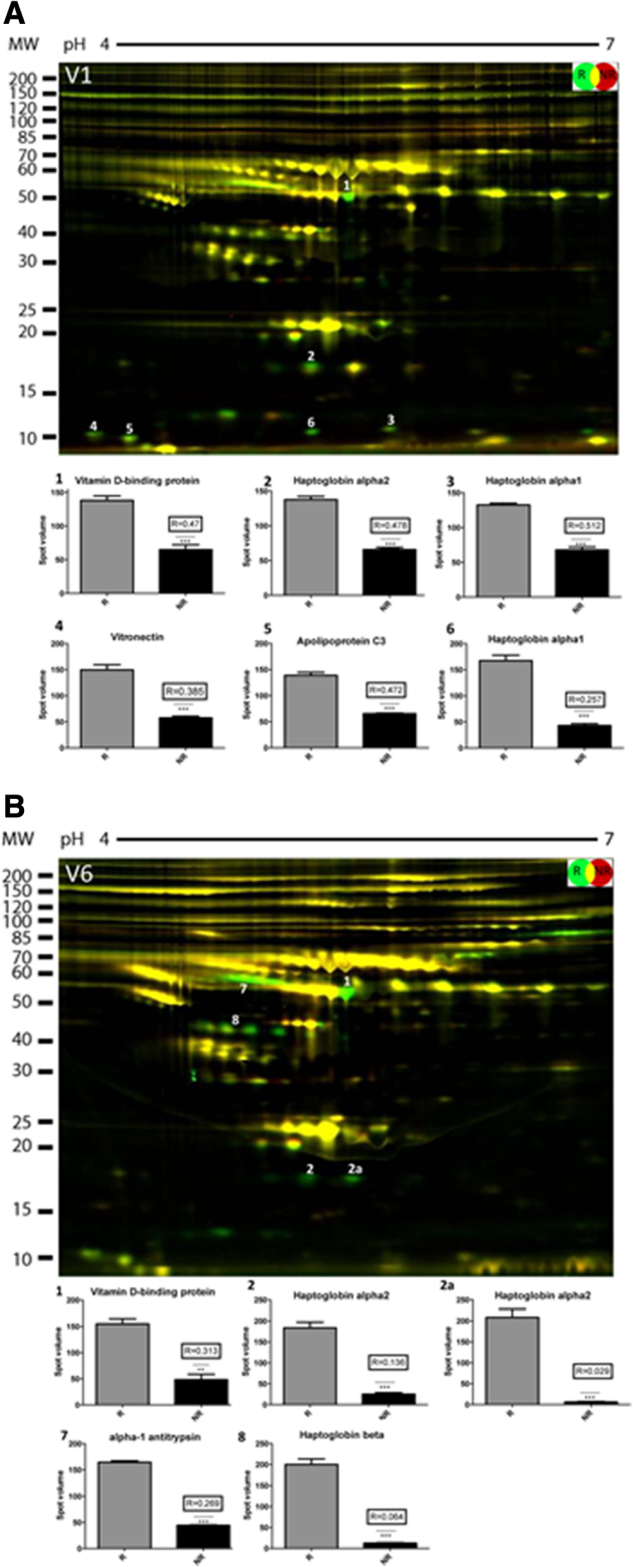
**Serum proteome profiles of responders and nonresponders to etanercept treatment in rheumatoid arthritis patients.** Serum samples of responders (*n* = 5) and nonresponders (*n* = 5) were pooled after immunodepletion and subjected to two-dimensional differential gel electrophoresis analysis. Protein spot identification in serum proteome profiles of responders (R) and nonresponders (NR) was performed by subsequent mass spectrometry. **(A)** Prior to initiation of etanercept treatment (V1), five proteins were found to be significantly upregulated in responders including vitamin D-binding protein (VDBP), haptoglobin (Hp)-α1, Hp-α2, vitronectin and apolipoprotein C-III. **(B)** After 6 months of therapy (V6), VDBP, Hp-α2, Hp-β and α1-antitrypsin were found at significantly elevated levels in responder sera. MW, molecular weight.

To explore proteome differences between sera of responders and nonresponders, and thus to identify potential predictors for etanercept treatment outcome, sera from responders (*n* = 5) and nonresponders (*n* = 5) obtained at study entry (V1) and at the end of study (V6) were subjected to serum depletion using the Human-14® column coupled to high-performance liquid chromatography to remove the 145 most abundant serum proteins. To assess the reproducibility of the protein depletion, the same sample was processed five times over the column and data reproducibility was monitored using 2D-DIGE (see Additional file 1). After optimization of the depletion protocol, serum samples from different patients and visits were subjected to serum depletion followed by protein concentration and 2D-DIGE. Proteins found to be differentially expressed were excised and subjected to in-gel digestion and MS analyses. A total of 180 spots were identified, which resulted in 55 nonredundant proteins (see Additional files 2, 3, 4 and 5). Examination of single protein spots revealed significant differences between the protein profiles of responders and nonresponders. At baseline, five proteins were found to be significantly upregulated in responders (Figure 2A; see Additional file 6). These proteins were identified as vitamin D-binding protein (VDBP), Hp-α1, Hp-α2, vitronectin and ApoC-III. ApoA-II and ApoC-II levels were also elevated, but only significant in a small proportion of the samples. After 6 months of etanercept treatment (V6), VDBP, Hp-α2, Hp-β and α1-antitrypsin were detected at significantly higher levels in responders (Figure 2B).

### Confirmation of 2D-DIGE results by western blot analysis

**Figure 3:**
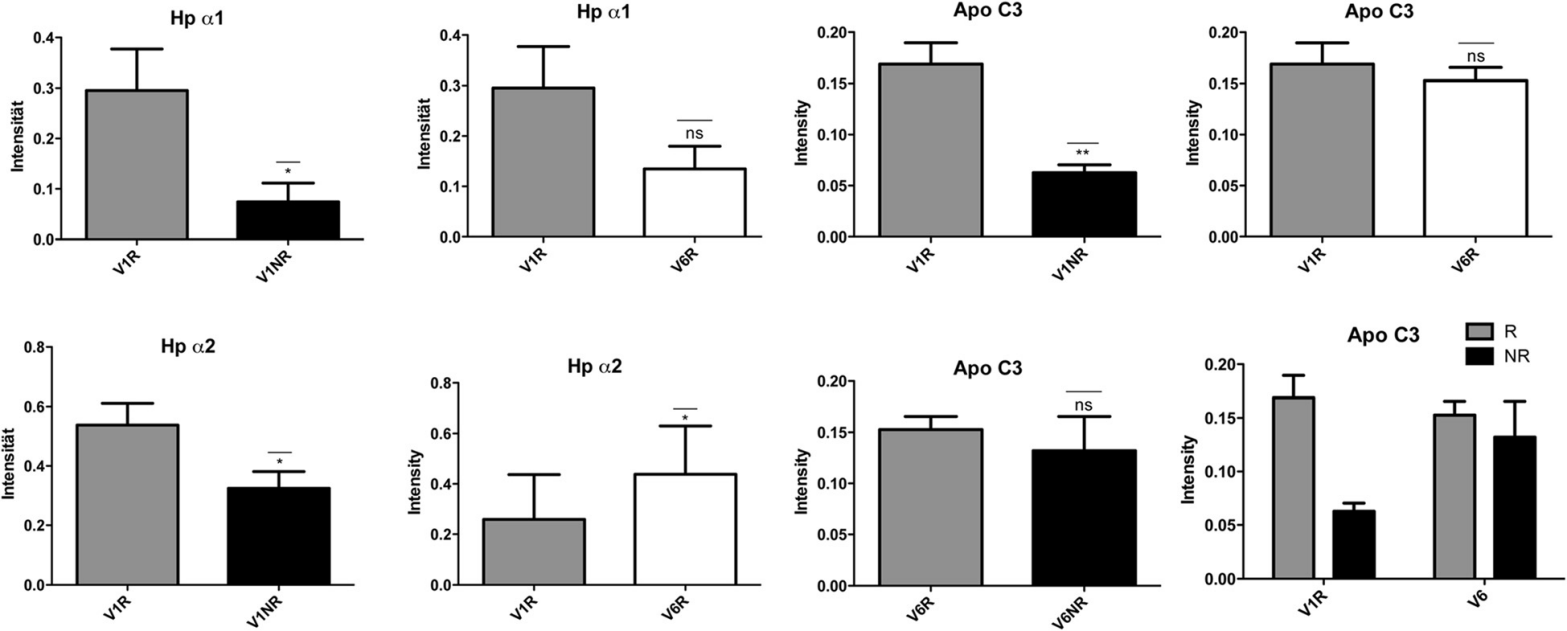
**Western blot analysis of responder and nonresponder sera.** To validate results of serum proteome profiling, western blot analyses for haptoglobin (Hp)-α1, Hp-α2, apolipoprotein (Apo)C-III and ApoA-II were carried out. Serum samples from responders (*n* = 5) and nonresponders (*n* = 5) were subjected to western blot analysis with antibodies against the mentioned proteins. At baseline (V1), responder sera revealed a significant upregulation of Hp-α1 and Hp-α2 band intensities in comparison with nonresponders. These results were also detected after 6 months of etanercept treatment (V6). In contrast, the ApoC-III upregulation in responders could only be confirmed at baseline (V1). ApoA-II levels were found to be significantly upregulated in responders only after 3 months (V5) and 6 months (V6) of therapy in comparison with nonresponders.

To confirm 2D-DIGE results, sera from responders (*n* = 5) and nonresponders (*n* = 5) were subjected to fluorescent WB analysis. As demonstrated in Figure 3, the data obtained by 2D-DIGE for Hp-α1, Hp-α2 and ApoC-III could be confirmed by WBs. In contrast, the higher levels of ApoA-II in responders compared with nonresponders seen in the 2D-DIGE analysis were found to hold true only for three patients out of five, and in two patients ApoA-II did not exhibit any significant differences in responders compared with nonresponders.

### Validation of 2D-DIGE results in a larger cohort of RA patients by western blot analysis

**Figure 4:**
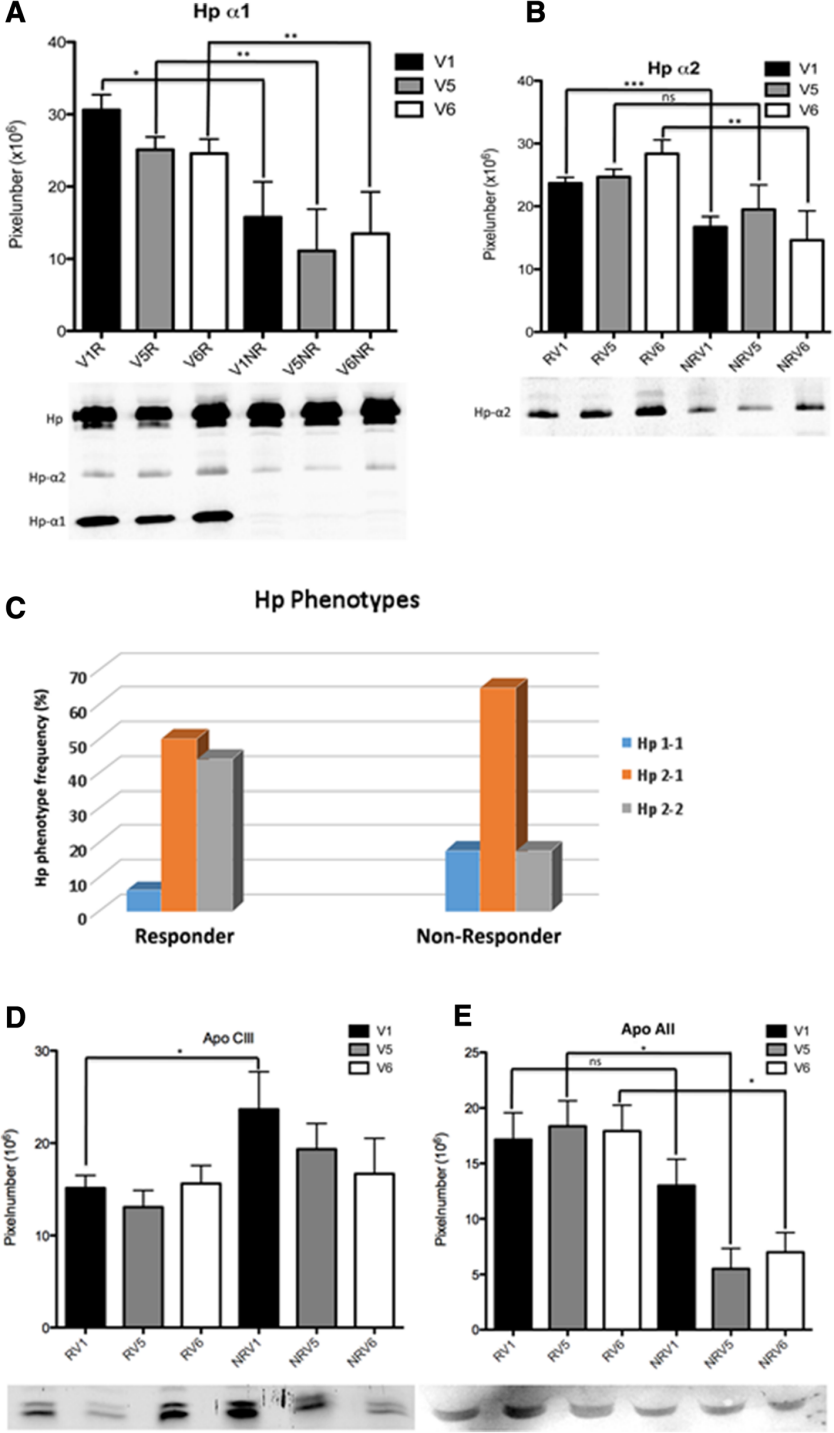
**Validation of results in a larger cohort of rheumatoid arthritis patients by western blot analysis.** To validate results of two-dimensional differential gel electrophoresis analyses, one-dimensional western blots were performed for haptoglobin (Hp)-α1, Hp-α2, apolipoprotein (Apo)A-II and ApoC-III in a larger cohort of rheumatoid arthritis patients (*n* = 31 responders and *n* = 19 nonresponders). **(A, B)** The statistical analysis of the western blot data confirmed the significant upregulation of Hp-α1 and Hp-α2 in responder (R) sera in comparison with nonresponder (NR) sera prior to etanercept treatment initiation (V1) (*P =* 0.0231, *P* = 0.0185, respectively) as well as after 6 months of therapy (V6) (*P =* 0.00445, *P* = 0.0038, respectively). **(C)** Interestingly, analysis of Hp phenotypes Hp 2-1, Hp 2-2 and Hp 1-1 revealed that the frequency of the phenotype Hp 2-2 was more common in R in comparison with NR, whereas the phenotype Hp 2-1 was found to be more common in NR. **(D)** In contrast to proteomics data, the western blot analysis in a larger patient cohort showed an upregulation of ApoC-III in NR sera prior to etanercept treatment (V1) in comparison with responders. **(E)** During the course of etanercept treatment, no more significant differences between R and NR could be detected. Upregulation of ApoA-II expression was detected in R in comparison with NR only after 3 months (V5) and 6 months (V6) of therapy.

To validate results of 2D-DIGE analyses in responders and nonresponders, one-dimensional WBs were performed for Hp-α1, Hp-α2, ApoA-II and ApoC-III in a larger cohort of RA patients (*n* = 31 responders and *n* = 19 nonresponders). Results confirmed significant upregulation of Hp-α1 and Hp-α2 in responder sera in comparison with nonresponder sera prior to etanercept treatment initiation (V1) (*P =* 0.0231, *P* = 0.0185, respectively) as well as after 6 months of therapy (V6) (*P =* 0.00445, *P* = 0.0038, respectively) (Figure 4A,B). Interestingly, analysis of Hp phenotypes revealed that the frequency of the phenotype Hp 2-2 was more common in responders, and the phenotype Hp 2-1 was found to be more common in nonresponders (Figure 4C).

In contrast to 2D-DIGE data, ApoC-III was found to be significantly upregulated in nonresponder sera prior to etanercept treatment (V1) (*P* = 0.0164) in comparison with responders (Figure 4D). During the course of etanercept treatment, results revealed no more significant differences between responders and nonresponders.

For ApoA-II, an upregulation of protein expression was detected in responders in comparison with nonresponders (Figure 4E). However, these results were only significant after 3 months (V5, *P* = 0.0114) and 6 months (V6, *P* = 0.0228) of therapy.

**Figure 5:**
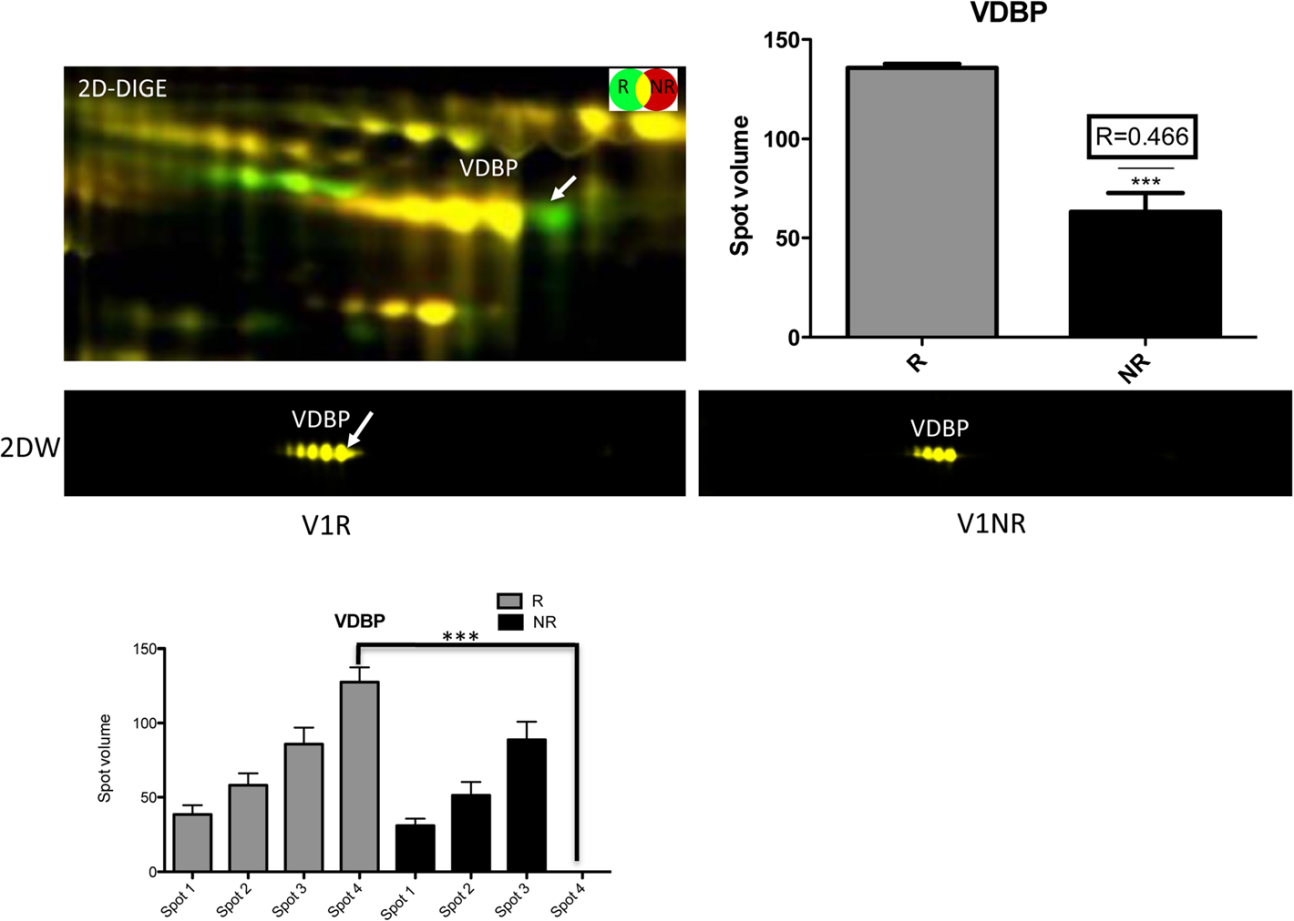
**Two-dimensional western blot analysis of vitamin D-binding protein isoforms in responders and nonresponders.** For analysis of vitamin D-binding protein (VDBP) expression, two-dimensional western blot (2D-WB) analysis was performed. Different isoforms of VDBP were detected at V1 in responders (R) and nonresponders (NR). 2D-WB revealed an additional VDBP isoform in R compared with NR, suggesting a posttranslational modification such as changes in glycosylation pattern. 2D-DIGE, two-dimensional differential gel electrophoresis.

Interestingly, in the case of VDBP the 2D-DIGE revealed the presence of an extra form of the proteins in responders compared with nonresponders, as evidenced by the spot pattern on the two-dimensional gel. The additional VDBP isoform seems to have a shift in isoelectric point suggesting a posttranslational modification such as changes in glycosylation pattern. To confirm these results, two-dimensional WBs were performed and demonstrated clearly that VDBP was present in four different forms in responder sera, but in only three isoforms in nonresponder sera (Figure 5).

## Discussion

In this study, identification of novel biomarkers as valid predictors of etanercept drug response in RA was successfully achieved by serum proteomic profiling in responders and nonresponders.

In RA, the introduction of TNFα inhibitors as biological agents has substantially improved the patient’s long-term outcome. Low disease activity or even disease remission now represents the ultimate therapeutic goal by adoption of a treat-to-target strategy to achieve tight disease control. However, several large clinical studies have demonstrated that up to 20 to 40% of RA patients do not respond to anti-TNFα biologicals. The prediction of individual response biological treatment has thus become a major clinical challenge. The need for a personalized therapeutic strategy in RA is stressed by the fact that early effective treatment has an enormous impact on patient’s overall prognosis for disease outcome. Furthermore, TNFα inhibitors may cause serious side effects and are cost-intensive in comparison with conventional DMARD therapies.

Several previous studies have analyzed clinical, laboratory and genetic factors as potential predictors of TNFα antagonist drug response: large cohort studies have revealed that older age, low functional status and concomitant prednisolone [22] as well as smoking, glucocorticoid use and worse physician assessment of disease activity at baseline [11,23] are negative predictors of clinical response to TNFα antagonists, whereas treatment with concomitant DMARDs and low disability were found to be associated with good response [24]. Heterogeneous results have been reported for the presence and titers of anti-cyclic citrullinated peptide antibodies [25,26]. Novel biomarkers such as IL-6 [25], IL-21 [27] and CD11c [28] as well as tissue biomarkers [29] have only been studied in small cohorts or experimental settings. Recent progress in defining genetic factors for response by analysis of single nucleotide polymorphisms [30-32] and genome-wide association studies [33] yielded promising results. Furthermore, serum proteome analysis studies have been performed previously in RA patients receiving biological therapies and detected differential expression of several small proteins including apolipoproteins [34-37]. However, none of these studies could clearly identify valid predictors for TNF-alpha antagonist drug response. Thus, none of these models has so far been translated into clinical application.

In this study we analyzed potential predictors of etanercept drug response by a clinical proteomics approach in sera of responders and nonresponders. Results revealed differential regulation of small serum protein expression between responders and nonresponders prior to the initiation of etanercept treatment and after 3 and 6 months of therapy. Significant upregulation of protein expression was detected in pretreatment samples of responders for Hp-α1, Hp-α2 and VDBP. In contrast, ApoC-III levels were found to be significantly upregulated in nonresponders prior to initiation of etanercept treatment. Furthermore, significant changes of these proteins were also detected during follow-up after 6 months of therapy between responders and nonresponders. By analyzing the impact of treatment duration (0, 3 and 6 months) on the protein level within the same patient group, no significant differences could be detected between V1, V5 and V6. Differences were only significant between responders and nonresponders. This finding suggests that there is no clear correlation between the treatment duration and the serum protein levels within the same group.

Haptoglobin is a polypeptide structurally consisting of two α chains (α1 or α2) and two β chains. Hp represents an acute phase protein that is induced via proinflammatory cytokines such as IL-6, IL-1 or TNFα. According to the presence of the α1 or α2 chains, three different phenotypes (Hp 1-1, Hp 2-1, Hp 2-2) could be distinguished [38]. Previous studies have revealed that Hp 2-2 phenotypes are genetically associated with inflammatory and autoimmune diseases [39]. Functionally, Hp binds free hemoglobin and thus represents a marker for hemolysis. Furthermore, Hp was shown previously to exert additional anti-oxidative, anti-inflammatory and immunoregulatory effects: owing to the structural similarity with 7S immunoglobulin, Hpα may suppress cellular immune responses. In addition, Hp was shown to restore homeostasis after inflammatory processes [40] and to bind on activated macrophages, thus leading to the inhibition of TNFα production [41]. In this study, responders were characterized by a significant upregulation of Hpα1 and Hpα2 production prior to the initiation and after 6 months of etanercept therapy in RA. These findings may support the hypothesis that Hpα chains may support anti-inflammatory effects of etanercept in RA. Responders may profit from upregulated Hpα chain expression in RA prior to and during the course of etanercept therapy. Furthermore, the Hp 2-2 phenotypes were found to be more common in responders, whereas Hp 2-1 were mainly detected in the nonresponder group. Upregulation of Hpα_1_ and Hpα_2_ expression and Hp phenotype thus may represent valid markers for prediction of response to etanercept therapy in RA.

ApoC-III is a small protein containing 79 amino acids that resides on the surface of lipoproteins. Physiological functions of ApoC-III include the inhibition of lipoprotein lipase and hepatic lipase. ApoC-III thus represents a major regulator of lipolysis. In consequence, elevated ApoC-III levels have been detected in patients with hypertriglyceridemia [42]. Furthermore, genetic variation studies have revealed that distinct ApoC-III haplotypes predispose for a higher risk of coronary heart disease and diabetes mellitus. In addition to these effects on lipolysis, there is emerging scientific evidence that ApoC-III exerts proinflammatory effects on both monocytes and endothelial cells during the pathogenesis of atherosclerosis: ApoC-III was previously shown to induce the expression of intercellular adhesion molecule-1 and vascular adhesion molecule-1 in endothelial cells [43]. Furthermore, ApoC-III was found to augment arterial inflammation by stimulating the adherence of peripheral monocytes to endothelial cells [44] and to initiate the transcription of proinflammatory cytokines via activation of nuclear factor-κB in monocytes [45]. In this study, significantly elevated ApoC-III serum levels were detected in the nonresponder group in comparison with responders prior to initiation of etanercept therapy. In correlation with previous findings concerning cardiovascular comorbidity, nonresponders in our study population were characterized by a higher (but nonsignificant) incidence of concomitant cardiovascular risk factors (Table 1). Upregulation of ApoC-III in nonresponders may counteract therapeutic effects of etanercept by antagonism of TNFα blockade and thus may be responsible for treatment failure in this RA patient group. This hypothesis may be supported by the finding that ApoC-III levels positively correlate with IL-6 serum levels at study entry (data not shown). This correlation was statistically not significant, however, perhaps due to the limited number of samples analyzed in this study.

VDBP is a multifunctional α_2_-globulin (50 to 52 kDa). Its main functional role is the binding and transport of vitamin D metabolites to effector cells. Owing to the fact that vitamin D3 is essential for bone metabolism and exerts immunomodulatory effects via inhibition of T-helper cell type 1 T-cell responses and proinflammatory cytokine action (IL-1, IL-6), VDBP is thought to support positive effects on both bone metabolism and immune system by induction of anti-inflammatory pathways [46]. Furthermore, VDBP is essential for G-actin binding to inhibit uncontrolled intravascular G-actin polymerization after cellular tissue damage, and thus to inhibit microembolism and organ dysfunction after cellular necrosis.

In responders, VDBP was detected at significantly elevated levels in this study. It is thus hypothesized that in responders VDBP and etanercept may exert synergistic effects on bone metabolism and immune function in RA. In this respect, responders characterized by elevated VDBP levels may profit from a higher vitamin D3 availability and G-actin binding capacity after cellular necrosis in comparison with nonresponders. This hypothesis may be supported by the finding of a negative correlation between VDBP and IL-6 serum levels at study entry (data not shown); however, the correlation analysis was again statistically not significant. The small number of serum samples analyzed and the large number of factors (cytokines, hormones and concomitant diseases) known to influence IL-6 serum levels may account for this finding.

Interestingly, a posttranslational modification of VDBP was detected in responders by two-dimensional WB as compared with nonresponders. The pattern of this modification suggests changes in glycosylation status for responders. Previous studies have demonstrated that glycosylation of VDBP leads to the production of macrophage activating factor via T-cell and B-cell associated enzymes [47]. Macrophage activating factor production was shown to induce proinflammatory, anti-angiogenic, anti-tumorous and anti-proliferative effects [48]. It is thus hypothesized that, in nonresponders characterized by glycosylated isoforms of VDBP, treatment failure of etanercept may be due to the overproduction of macrophage activating factor. However, this hypothesis still has to be analyzed and validated in future experimental studies.

In this study, several biomarkers for response to etanercept treatment could be identified by application of a clinical proteomics approach. However, a small sample size represents a limitation of the study. Furthermore, limitations of the clinical proteomics approach in identifying biomarkers are demonstrated and underline the necessity of proteomics data validation.

## Conclusions

By application of a clinical proteomics approach, our study demonstrates that small serum proteins such as Hp-α1, Hp-α2, VDBP and ApoC-III may serve as valid predictors for etanercept drug response in RA. Further studies in larger and independent cohorts are required to confirm the study results and to develop the best model with the greatest predictive accuracy for treatment response to etanercept in RA.

## Additional files

Additional file 1:
**A figure showing serum depletion by immunoaffinity chromatography.** Serum samples were cleared of the 14 most abundant major proteins by immunoaffinity chromatography using Human-14® immunoaffinity columns according to the manufacturer’s protocol. Serum proteome gels before depletion (upper panel), depleted high abundant proteins (lower panel left) and low abundant proteins (lower panel right) are demonstrated. Additional file 2:
**Figure showing 2D-DIGE analysis of RA patients’ sera.** For 2D-DIGE, 50 μg proteins (from each sample) labeled with the appropriate dye were loaded on an 11 cm immobilized pH gradient strip with a linear pH gradient pI of 5 to 8 for isoelectric focusing, 12% SDS-polyacrylamide gels were used for the SDS-PAGE. The gels were compared with Delta 2D (Decodon). Protein identification was carried out using MS and data bank search. Blue spots, proteins in responder (R) sera; orange spots, proteins in nonresponder (NR) sera; black spots, proteins present in both R and NR sera. **(A)** 2D-DIGE from sera taken prior to etanercept treatment (V1), **(B)** 2D-DIGE from sera taken 3 months (V5) after start of treatment, **(C)** 2D-DIGE from sera obtained at 6 months (V6) after initiation of etanercept therapy. Additional file 3:
**A table presenting MS analysis and protein identification of differentially expressed proteins at V1.**
Additional file 4:
**A table presenting MS analysis and protein identification of differentially expressed proteins at V6.**
Additional file 5:
**A table presenting MS analysis and protein identification of differentially expressed proteins at V5.**
Additional file 6:
**A table presenting spot quantification at baseline (V1) in responders and nonresponders.**

## Abbreviations

Apo: apolipoprotein
CHAPS: 3-((3-cholamidopropyl)-dimethylammonio)-1-propane sulfonate
CRP: C-reactive protein
DAS28: Disease Activity Score in 28 joints
2-DE: two-dimensional gel electrophoresis
2D-DIGE: two dimensional differential gel electrophoresis
DMARD: disease-modifying antirheumatic drug
DTT: dithiothreitol
Hp: haptoglobin
IL: interleukin
MS: mass spectrometry
RA: rheumatoid arthritis
TNFα: tumor necrosis factor alpha
V: visit
VDBP: vitamin D-binding protein
WB: western blot
