# Supplementary figures and images for: Haptoglobin-α1, -α2, vitamin D-binding protein and apolipoprotein C-III as predictors of etanercept drug response in rheumatoid arthritis
Source: Arthritis Res Ther. 2015 Mar 6;17(1):45. doi: 10.1186/s13075-015-0553-1 (PMC4383078; doi:10.1186/s13075-015-0553-1)

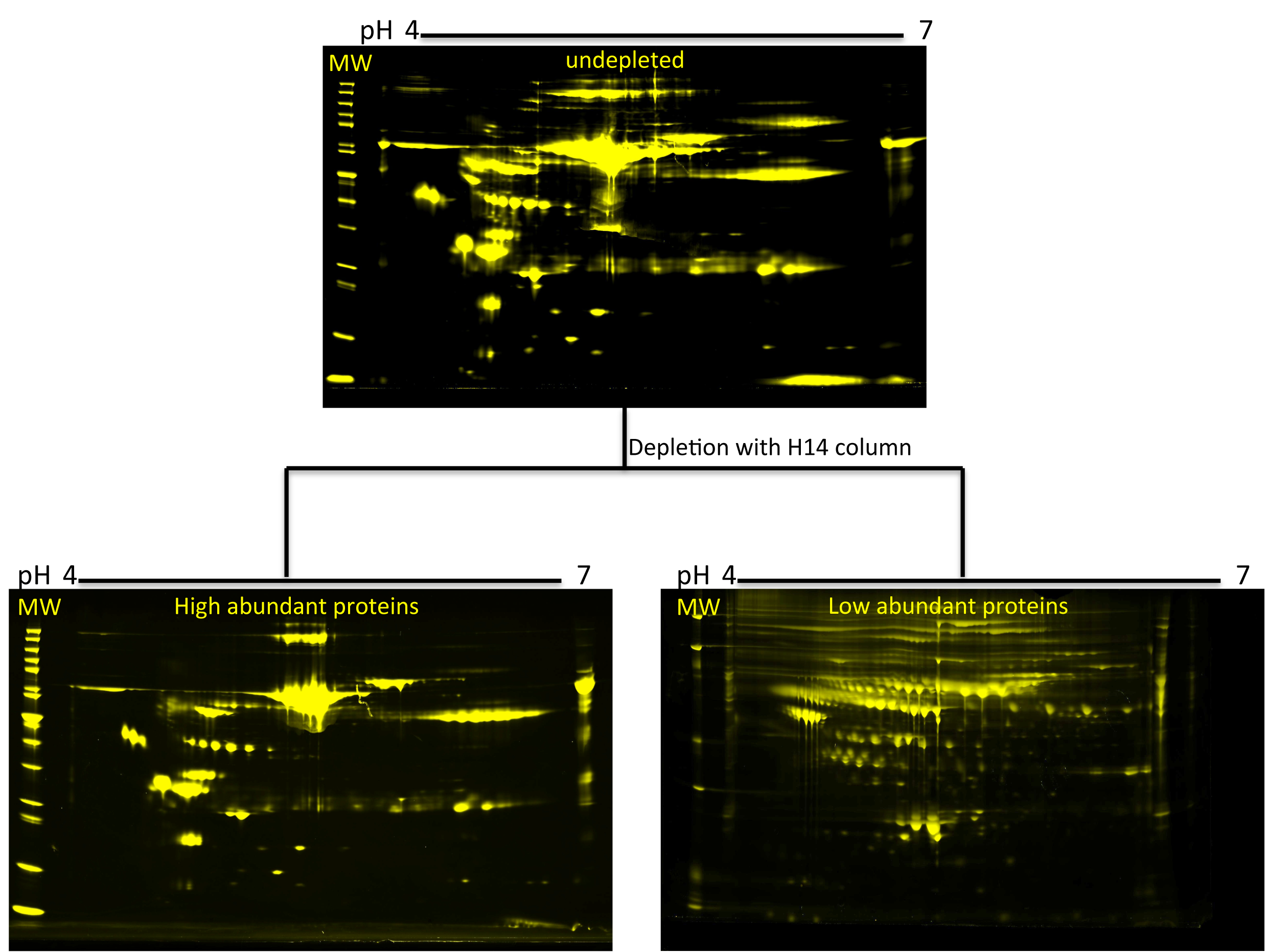

Supplement: Additional file 1: — A figure showing serum depletion by immunoaffinity chromatography. Serum samples were cleared of the 14 most abundant major proteins by immunoaffinity chromatography using Human-14® immunoaffinity columns according to the manufacturer’s protocol. Serum proteome gels before depletion (upper panel), depleted high abundant proteins (lower panel left) and low abundant proteins (lower panel right) are demonstrated. [file 13075_2015_553_MOESM1_ESM.tiff]

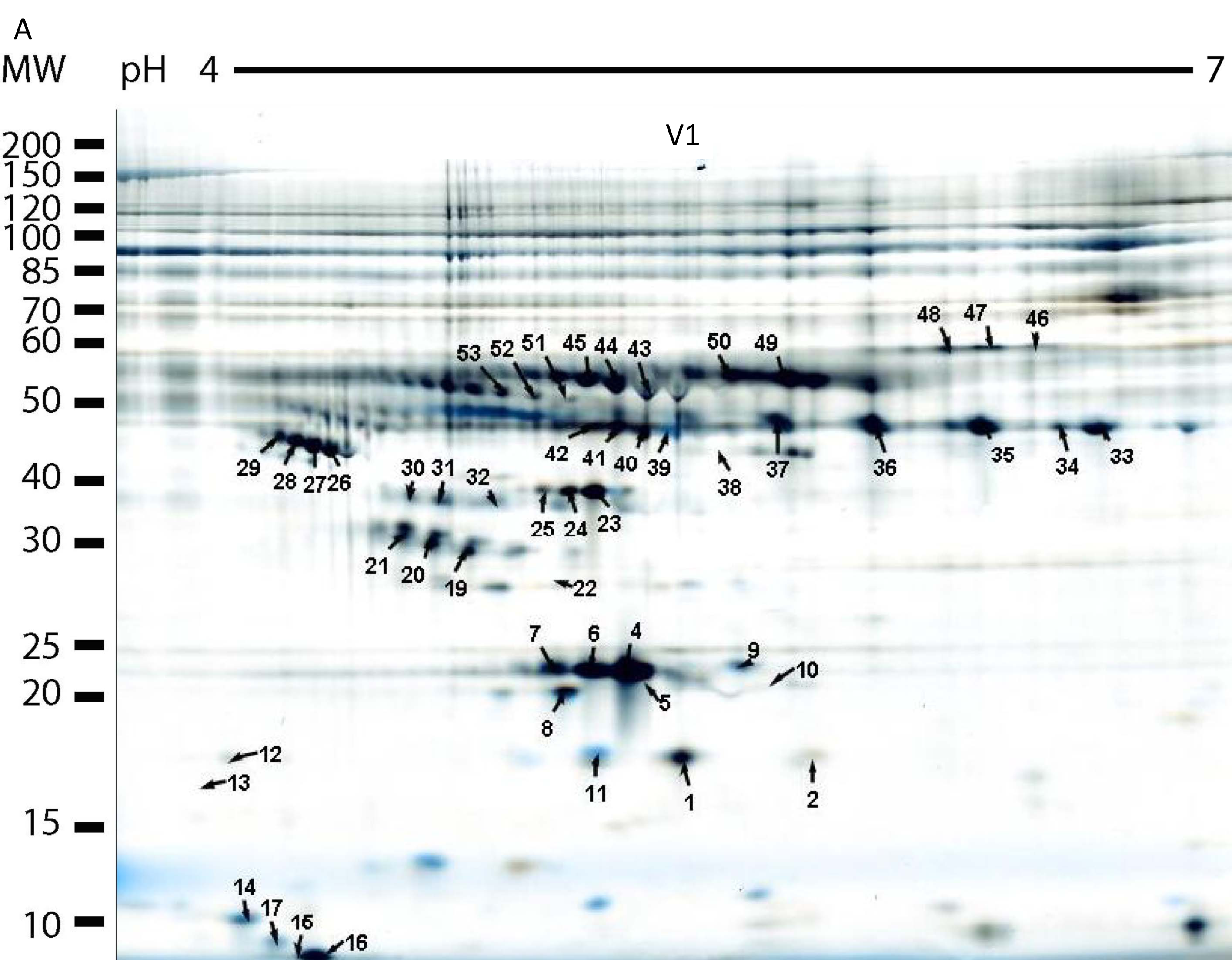

Supplement: Additional file 2: — Figure showing 2D-DIGE analysis of RA patients’ sera. For 2D-DIGE, 50 μg proteins (from each sample) labeled with the appropriate dye were loaded on an 11 cm immobilized pH gradient strip with a linear pH gradient pI of 5 to 8 for isoelectric focusing, 12% SDS-polyacrylamide gels were used for the SDS-PAGE. The gels were compared with Delta 2D (Decodon). Protein identification was carried out using MS and data bank search. Blue spots, proteins in responder (R) sera; orange spots, proteins in nonresponder (NR) sera; black spots, proteins present in both R and NR sera. (A) 2D-DIGE from sera taken prior to etanercept treatment (V1), (B) 2D-DIGE from sera taken 3 months (V5) after start of treatment, (C) 2D-DIGE from sera obtained at 6 months (V6) after initiation of etanercept therapy. [file 13075_2015_553_MOESM2_ESM.zip › add2/6750590791251850_add2.tiff]

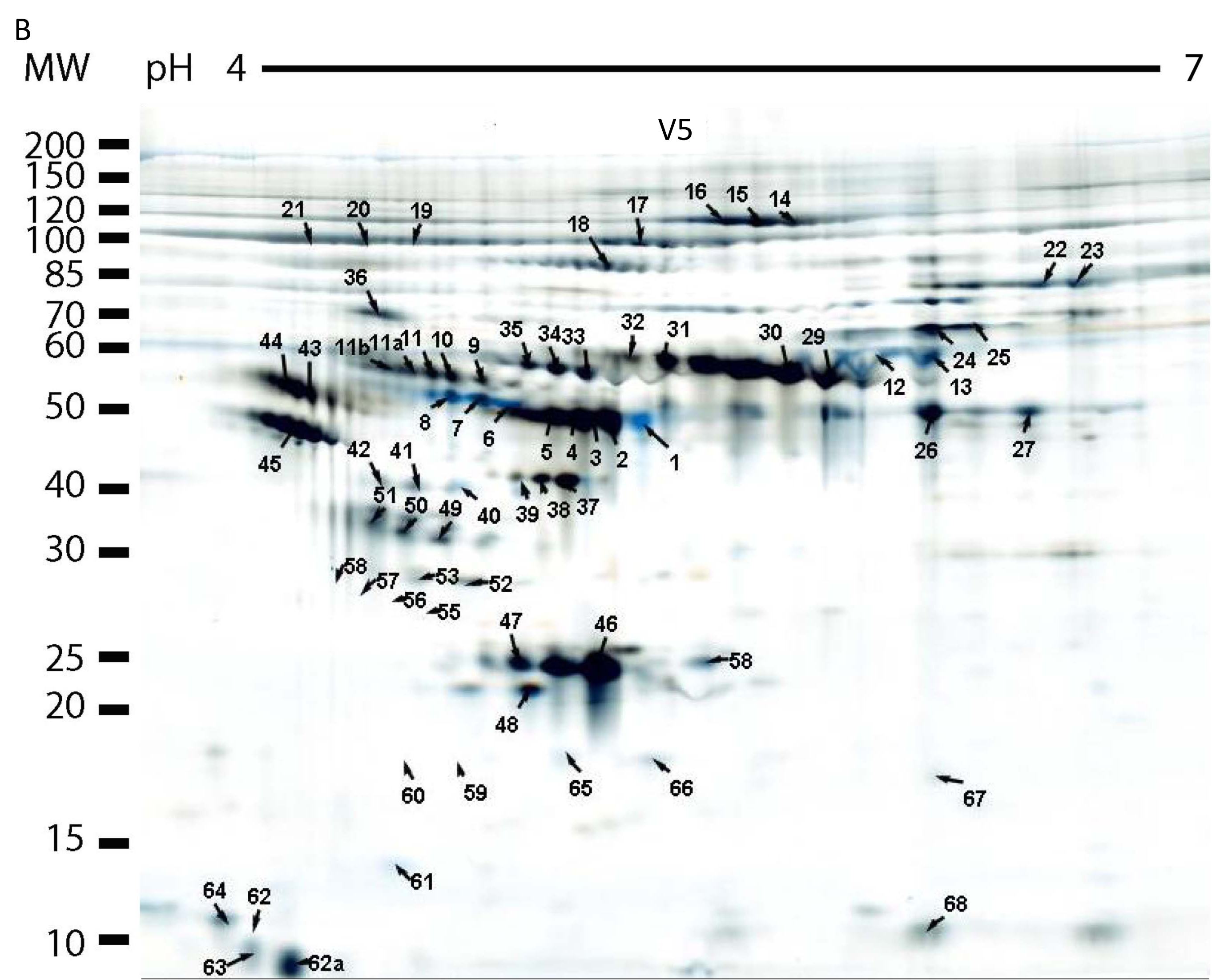

Supplement: Additional file 2: — Figure showing 2D-DIGE analysis of RA patients’ sera. For 2D-DIGE, 50 μg proteins (from each sample) labeled with the appropriate dye were loaded on an 11 cm immobilized pH gradient strip with a linear pH gradient pI of 5 to 8 for isoelectric focusing, 12% SDS-polyacrylamide gels were used for the SDS-PAGE. The gels were compared with Delta 2D (Decodon). Protein identification was carried out using MS and data bank search. Blue spots, proteins in responder (R) sera; orange spots, proteins in nonresponder (NR) sera; black spots, proteins present in both R and NR sera. (A) 2D-DIGE from sera taken prior to etanercept treatment (V1), (B) 2D-DIGE from sera taken 3 months (V5) after start of treatment, (C) 2D-DIGE from sera obtained at 6 months (V6) after initiation of etanercept therapy. [file 13075_2015_553_MOESM2_ESM.zip › add2/6750590791251850_add3.tiff]

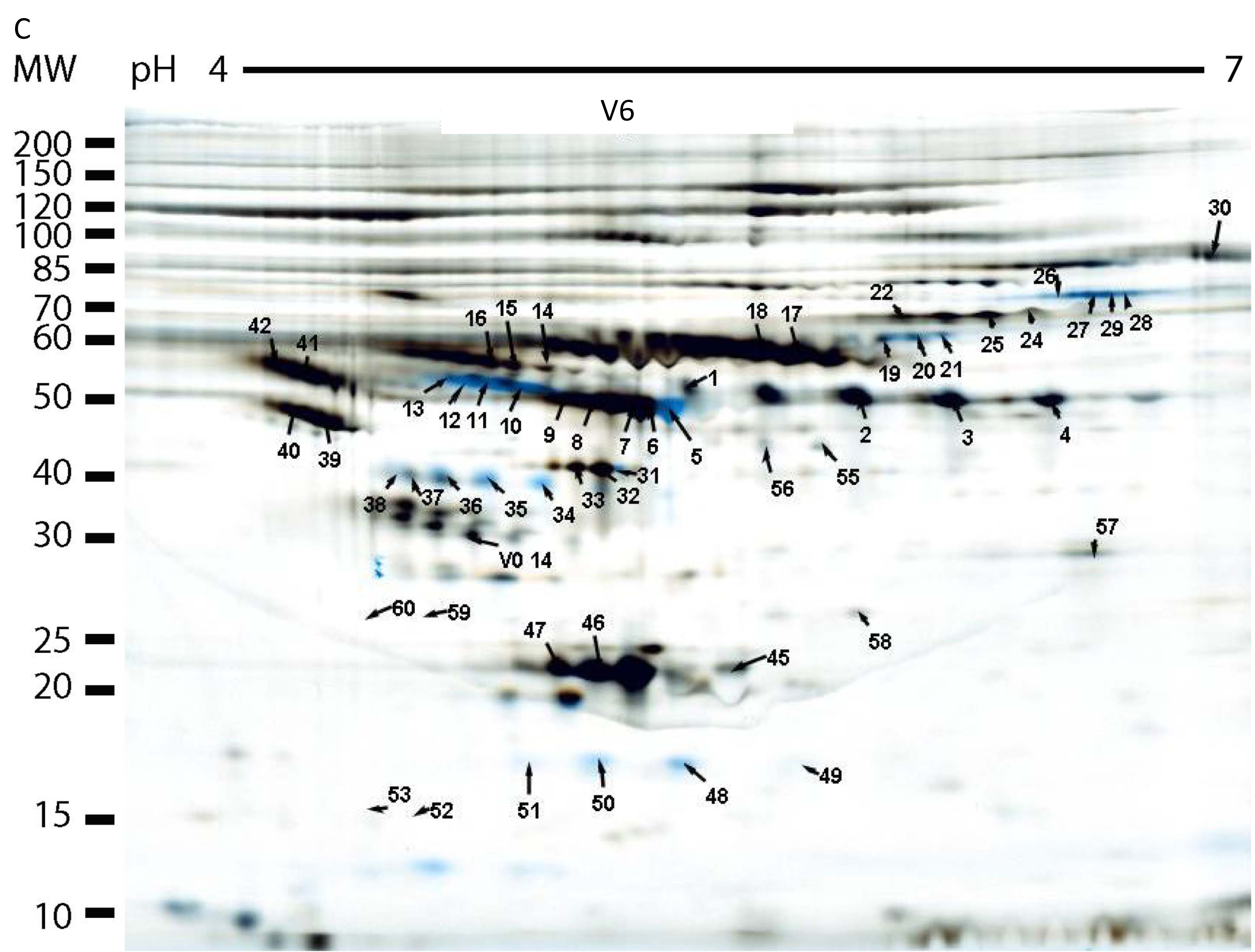

Supplement: Additional file 2: — Figure showing 2D-DIGE analysis of RA patients’ sera. For 2D-DIGE, 50 μg proteins (from each sample) labeled with the appropriate dye were loaded on an 11 cm immobilized pH gradient strip with a linear pH gradient pI of 5 to 8 for isoelectric focusing, 12% SDS-polyacrylamide gels were used for the SDS-PAGE. The gels were compared with Delta 2D (Decodon). Protein identification was carried out using MS and data bank search. Blue spots, proteins in responder (R) sera; orange spots, proteins in nonresponder (NR) sera; black spots, proteins present in both R and NR sera. (A) 2D-DIGE from sera taken prior to etanercept treatment (V1), (B) 2D-DIGE from sera taken 3 months (V5) after start of treatment, (C) 2D-DIGE from sera obtained at 6 months (V6) after initiation of etanercept therapy. [file 13075_2015_553_MOESM2_ESM.zip › add2/6750590791251850_add4.tiff]
